# From Policy to Practice: Advancing Institutional Readiness in Sustainable Food Systems

**DOI:** 10.3390/nu18081300

**Published:** 2026-04-20

**Authors:** Mariusz Jaworski

**Affiliations:** Department of Education and Research in Health Sciences, Faculty of Health Science, Medical University of Warsaw, 00-581 Warsaw, Poland; mariusz.jaworski@wum.edu.pl

The transformation of food systems toward greater sustainability has become one of the key challenges in public health. This is driven by the co-occurrence of global issues such as climate change, non-communicable diseases (e.g., obesity, type 2 diabetes), and environmental degradation [1,2]. Although a growing body of research highlights the health and environmental benefits of sustainable dietary patterns [3,4,5], their practical implementation remains uneven and often limited. The gap between intended goals and their real-world implementation represents one of the most significant challenges in contemporary dietetics and public health [6,7].

Over the past decade, policy frameworks at international and national levels have increasingly emphasized the importance of sustainable food systems. However, their existence alone does not guarantee effective implementation. In practice, institutions responsible for implementing nutrition-related policies, including public food service institutions and local government bodies, operate under complex conditions shaped by economic constraints, legal requirements, and organizational realities. As a result, the success of sustainability-oriented interventions depends not only on the strength of the evidence base but also on the ability of institutions to function within these constraints in ways that are sensitive to local context [8].

This Special Issue, Future Prospects for Sustaining a Healthier Food System, makes an important contribution to this discussion by presenting diverse perspectives on sustainable food systems across multiple levels of analysis. The included studies illustrate the multidimensional nature of the transition toward sustainability. Some contributions focus on interventions implemented in real-world institutional settings, highlighting both the potential and limitations of context-sensitive approaches to shaping food environments and reducing food waste. Others examine behavioral determinants, including the persistent gap between dietary intentions and actual behaviors. Additional studies address structural characteristics of food systems, consumer decision-making processes, and emerging technological and nutritional innovations. Taken together, these findings suggest that advancing sustainable food systems requires not only generating evidence but also understanding how such evidence can be effectively implemented in real-world contexts.

The analysis of existing evidence suggests that the key challenge lies not only in identifying effective solutions, but also in the capacity of institutions to operationalize them [9]. In this context, the concept of institutional readiness is proposed as a useful perspective for understanding the implementation of sustainable food systems. Institutional readiness refers to the extent to which organizations are able to translate normative commitments into effective and context-appropriate practices [8].

This perspective can be conceptualized as the result of interactions between three interrelated dimensions. The first, normative orientation, reflects the extent to which institutions adopt and internalize goals related to health, sustainability, and food system transformation. The second, structural constraints, encompasses economic, legal, and organizational conditions that shape implementation feasibility, such as budget limitations, public procurement regulations, and market availability. The third, adaptive capacity, refers to the ability of institutions and their actors to navigate these constraints through local strategies, capacity-building, and cross-sector collaboration, including collaboration between public administration and the scientific community. Institutional readiness emerges from the dynamic interplay of these dimensions, and their misalignment may lead to implementation gaps, even in the presence of strong policy support.

This perspective shifts the focus from whether institutions declare support for sustainable food systems toward whether they are adequately prepared to implement them. It also highlights that discrepancies between intended goals and actual practices should not be viewed solely as barriers, but as inherent features of the context in which interventions are implemented. From this perspective, effective strategies are those that are not only evidence-based, but also feasible, adaptable, and responsive to local conditions.

This approach aligns with broader developments in implementation science, which emphasize the need to move beyond efficacy-focused research toward understanding how interventions function in real-world systems [8]. In the context of sustainable food systems, this requires greater attention to institutional processes, governance structures, and the practical realities of food provision. It also calls for interdisciplinary approaches integrating nutrition science, public health, policy studies, and behavioral research.

The findings presented in this Special Issue support the relevance of this perspective. They indicate that reducing the gap between policy and practice requires not only stronger evidence, but also a deeper understanding of institutional contexts and organizational capacities. Future research should therefore focus on implementation processes, including mechanisms that enable better alignment between normative goals and structural realities. There is also a need to design and evaluate interventions that explicitly account for resource constraints, organizational diversity, and the complexity of food environments.

From a policy perspective, strengthening institutional readiness may involve revisiting public procurement frameworks, supporting capacity-building initiatives, and enhancing collaboration among stakeholders. In particular, developing sustainable and structured mechanisms of collaboration between public administration and the scientific community may facilitate better alignment between evidence-based recommendations and real-world implementation conditions. From a research perspective, this requires methodological approaches that combine quantitative data with in-depth qualitative insights into institutional functioning. Ultimately, the transformation of food systems will depend not only on what is recommended, but on what is feasible within the systems responsible for delivering food to populations.

This Special Issue highlights the importance of moving beyond a focus on dietary recommendations toward a more comprehensive understanding of implementation processes in context. The concept of institutional readiness contributes to an emerging discourse that recognizes the central role of institutions in shaping the feasibility and sustainability of food system transformation. Bridging the gap between policy and practice is not merely a technical challenge, but a systemic one, requiring alignment between goals, constraints, and capacities in real-world settings.
